# Smad proteins differentially regulate obesity-induced glucose and lipid abnormalities and inflammation via class-specific control of AMPK-related kinase MPK38/MELK activity

**DOI:** 10.1038/s41419-018-0489-x

**Published:** 2018-04-27

**Authors:** Hyun-A Seong, Ravi Manoharan, Hyunjung Ha

**Affiliations:** 1 Department of Biochemistry, School of Biological Sciences, Chungbuk National University, Cheongju, 28644 Republic of Korea; 2 Department of Biochemistry, University of Madras, Guindy Campus, Chennai, 600025 India

## Abstract

Smad proteins have been implicated in metabolic processes, but little is known about how they regulate metabolism. Because Smad 2, 3, 4, and 7 have previously been shown to interact with murine protein serine–threonine kinase 38 (MPK38), an AMP‐activated protein kinase (AMPK)-related kinase that has been implicated in obesity-associated metabolic defects, we investigated whether Smad proteins regulate metabolic processes via MPK38. Smads2/3/4 increased, but Smad7 decreased, MPK38-mediated apoptosis signal-regulating kinase-1 (ASK1)/transforming growth factor-β (TGF-β)/p53 signaling. However, MPK38-mediated phosphorylation-defective Smad mutants (Smad2 S245A, Smad3 S204A, Smad4 S343A, and Smad7 T96A) had no such effect. In addition, Smads2/3/4 increased, but Smad7 decreased, the stability of MPK38. Consistent with this, Smads2/3/4 attenuated complex formation between MPK38 and its negative regulator thioredoxin (Trx), whereas Smad7 increased this complex formation. However, an opposite effect was observed on complex formation between MPK38 and its positive regulator zinc-finger-like protein 9 (ZPR9). When Smads were overexpressed in high-fat diet (HFD)-fed obese mice using an adenoviral delivery system, Smads2/3/4 improved, but Smad7 worsened, obesity-associated metabolic parameters and inflammation in a MPK38 phosphorylation-dependent manner. These findings suggest that Smad proteins have class-specific impacts on obesity-associated metabolism by differentially regulating MPK38 activity in diet-induced obese mice.

## Introduction

The identification of a growing list of intracellular kinases that phosphorylate Smad proteins suggests that the transforming growth factor-β (TGF-β)/Smad signaling pathway cross-talks with a variety of other intracellular signaling pathways^1^. The TGF-β signaling pathway regulates a broad range of cellular processes, which include cell proliferation, differentiation, apoptosis, migration, extracellular matrix remodeling, immune functions, and tumor metastasis. This occurs through the combined use of TGF-β signaling pathway components, such as Smads and Smad-interacting transcription factors, cross-talk with other intracellular signaling pathways, and the ability of TGF-β receptors to activate other signaling modules^2–6^. Many studies have shown that Smads are phosphorylated by multiple intracellular kinases, including mitogen-activated protein kinases, Ca^2+^/calmodulin-dependent kinase II, cyclin-dependent kinase (CDK), protein kinase C, G protein-coupled receptor kinase 2, extracellular signal-regulated kinase, apoptosis signal-regulating kinase-1 (ASK1), and murine protein serine–threonine kinase 38 (MPK38)/maternal embryonic leucine zipper kinase (MELK)^1,7,8^, suggesting that the TGF-β pathway is closely integrated with other intracellular signaling pathways to achieve tightly regulated TGF-β responses. However, most of these studies have focused on the regulatory role of Smad phosphorylation in the TGF-β signaling pathway. Additional studies are required to investigate the effect of Smad proteins on the activity of these interacting kinases in order to decipher the molecular interplay between TGF-β and other intracellular signaling pathways.

MPK38/MELK, an AMP‐activated protein kinase (AMPK)-related kinase, has been shown to mediate various cellular functions, including proliferation, spliceosome assembly, gene expression, carcinogenesis, apoptosis, and metabolism^9–13^, although its exact physiological functions still remain to be determined. MPK38 and its interacting partner Smad3 have recently been shown to serve as components of a multi-protein complex linking ASK1 and TGF-β signaling pathways, which are involved in glucose and lipid metabolism in mice, and to contribute to the activation of ASK1 signaling via a direct interaction with ASK1^8,11^. TGF-β1 was previously reported to positively regulate the 3-phosphoinositide-dependent protein kinase-1 (PDK1)/AKT1 pathway^14^, although PDK1 was shown to inhibit TGF-β signaling through direct interactions with Smads^15^. These findings suggest potential roles of Smads in the regulation of key kinases involved in intracellular signaling pathways that are integrated with TGF-β signaling.

Recent discoveries have shed some light on the important role that TGF-β signaling plays in adipose physiology and metabolism^16–18^. Smad3 deficiency in mice resulted in improved glucose tolerance and insulin sensitivity, accompanied by reduced white adipose tissue (WAT) mass and browning. The associated increase in mitochondrial biogenesis resulted in the dissipation of the excess energy stored in WAT by thermogenesis^16,17^. Higher TGF-β1 in humans has been shown to positively correlate with greater adiposity and a poor metabolic profile, and to negatively correlate with fitness^17^. Several recent studies have demonstrated that TGF-β signaling regulates insulin gene transcription in pancreatic β cells^19^. Moreover, the Smad3 gene was identified in a genome-wide association study for type 2 diabetes risk^20^. These findings implicate Smad3 as a potential target for the treatment of obesity and its associated disorders. Conversely, targeted disruption of Smad2 in mouse pancreatic β cells caused islet cell hyperplasia and impaired insulin secretion by attenuating ATP-sensitive K+ channel activity^21^. However, inhibition of Smad4 in pancreatic β cells conferred minor but significant improvements in blood glucose and glucose tolerance in high-fat diet (HFD)-induced obese mice^22^. Nevertheless, the molecular mechanisms involved in the regulation of metabolic homeostasis by TGF-β signaling remain poorly understood.

In this study, we show that there are direct physical and functional interactions between MPK38 and Smads (Smad2, 3, 4, and 7). Smads2/3/4 stimulate MPK38-dependent ASK1/TGF-β/p53 signaling pathways, whereas Smad7 inhibits these signaling pathways through differential regulation of MPK38 activity. Furthermore, overexpression of Smads2/3/4 improves, whereas Smad7 overexpression worsens, obesity-associated metabolic parameters by differentially regulating MPK38 activity in HFD-induced obese mice.

## Results

### MPK38 kinase activity is increased by Smads2/3/4 but decreased by Smad7

**Fig. 1:**
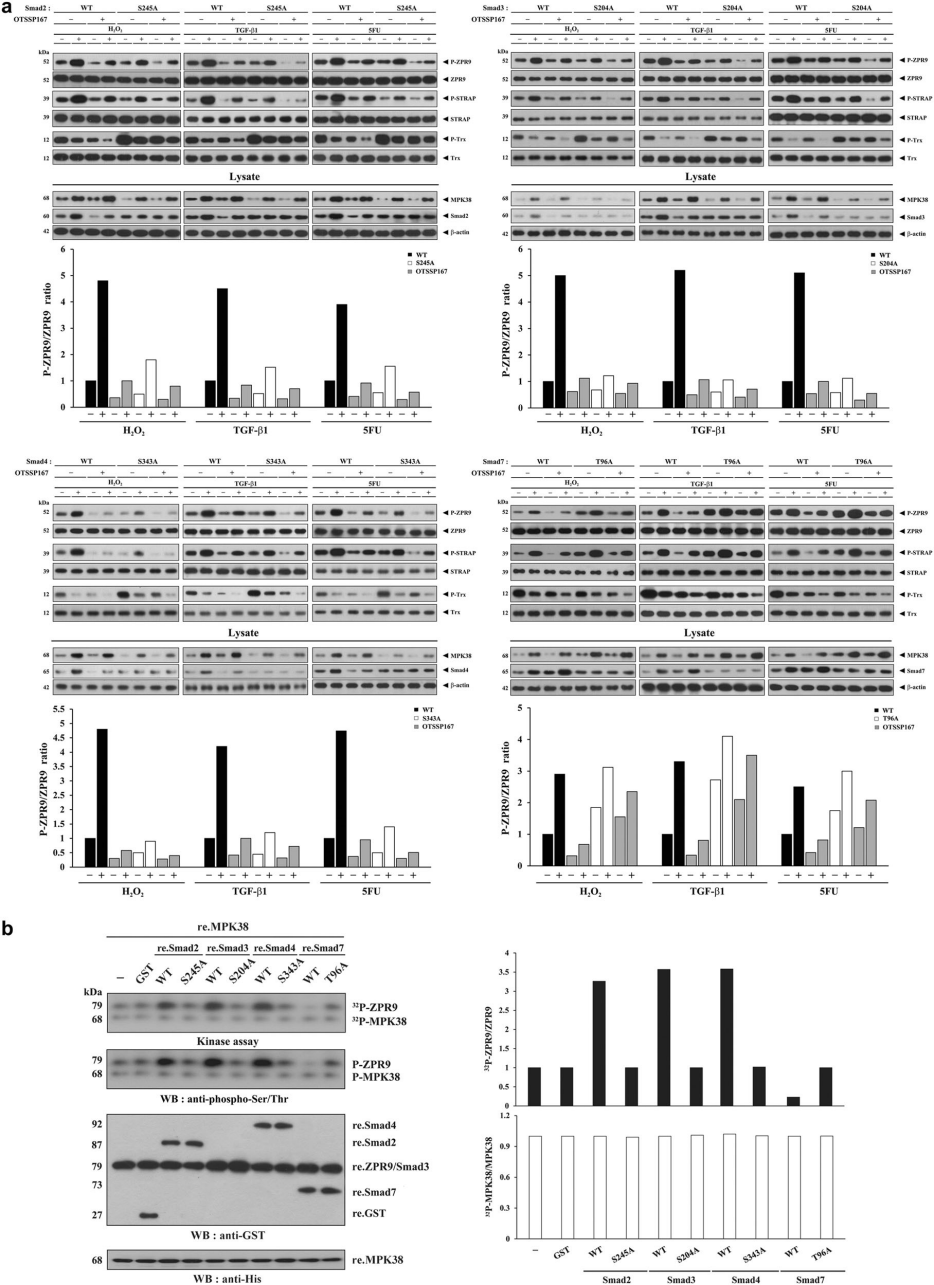
Differential regulation of MPK38 kinase activity by Smad proteins. **a** To assess the effects of wild-type (WT) and MPK38-mediated phosphorylation-defective Smad mutants (Smad2 S245A, Smad3 S204A, Smad4 S343A, and Smad7 T96A) on MPK38 kinase activity, WT and CRISPR/Cas9 Smad knock-in HEK293 cells (S245A, S204A, S343A, and T96A) were incubated with (+) or without (−) OTSSP167 (1 μM, 2 h), a potent MPK38 inhibitor, and then treated with or without the following stimuli: H_2_O_2_ (2 mM, 30 min), TGF‐β1 (2 ng/ml, 20 h), or 5FU (0.38 mM, 30 h). The cell lysates were subjected to immunoprecipitation with antibodies for ZPR9, STRAP, and Trx, followed by immunoblot analysis using anti-phospho-specific antibodies^23,24,34^ for ZPR9 Thr^252^, STRAP Ser^188^, and Trx Thr^76^. The protein levels of MPK38 and Smads in cell lysates were examined with anti-MPK38 and anti-Smads antibodies. **b** Recombinant MPK38 proteins (~5 μg) were screened in in vitro kinase assays using recombinant ZPR9 (~5 μg) as a substrate in the presence of recombinant WT and MPK38-mediated phosphorylation-defective Smad mutants (~0.8 μg each). Ratios of P-ZPR9/ZPR9 **a**, ^32^P-ZPR9/ZPR9 **b**, and ^32^P-MPK38/MPK38 **b** were determined by density analysis of the bands, and the fold-increase relative to the untreated WT controls **a** or control lacking Smad expression **b** is presented for each protein. ^32^P, ^32^P incorporation; P, phosphorylated; re., recombinant

Given that MPK38 interacts with and phosphorylates Smad proteins, leading to the activation of TGF-β signaling^7^, we reasoned that Smad proteins would affect MPK38 activity through direct interaction and phosphorylation. To assess this possibility, we performed immunoblot analysis using CRISPR/Cas9-mediated Smad knock-in (KI) HEK293 cells (Smad2 S245A, Smad3 S204A, Smad4 S343A, and Smad7 T96A), which are defective in MPK38-mediated phosphorylation^7^, to determine the endogenous kinase activity of MPK38 in the presence or absence of ASK1/TGF-β/p53 signals, including H_2_O_2_, TGF-β1, and 5-fluorouracil (5FU). The endogenous kinase activity of MPK38 was markedly lower in the S245A, S204A, and S343A KI cells when compared with wild-type control cells, whereas T96A KI cells had higher MPK38 kinase activity (Fig. 1a). These results were corroborated by inhibition of the kinase activity of MPK38 using a potent MPK38 inhibitor OTSSP167 (Fig. 1a)^23^. We also analyzed the effects of phosphorylation of Smad isoforms by MPK38 in the regulation of MPK38 kinase activity using in vitro kinase assays with recombinant wild-type and MPK38-mediated phosphorylation-defective Smad mutants. The MPK38 kinase activity was increased by recombinant wild-type Smads2/3/4, but decreased by recombinant wild-type Smad7. However, the recombinant MPK38-mediated phosphorylation-defective Smad mutants had no such effects (Fig. 1b). These findings indicate that Smad proteins differentially regulate the kinase activity of MPK38, resulting in specific Smad-mediated regulation of coordinate MPK38-induced ASK1/TGF-β/p53 signaling.

### MPK38-dependent ASK1/TGF-β/p53-mediated transcription, apoptosis, and signaling activation are increased by Smads2/3/4 but decreased by Smad7

**Fig. 2:**
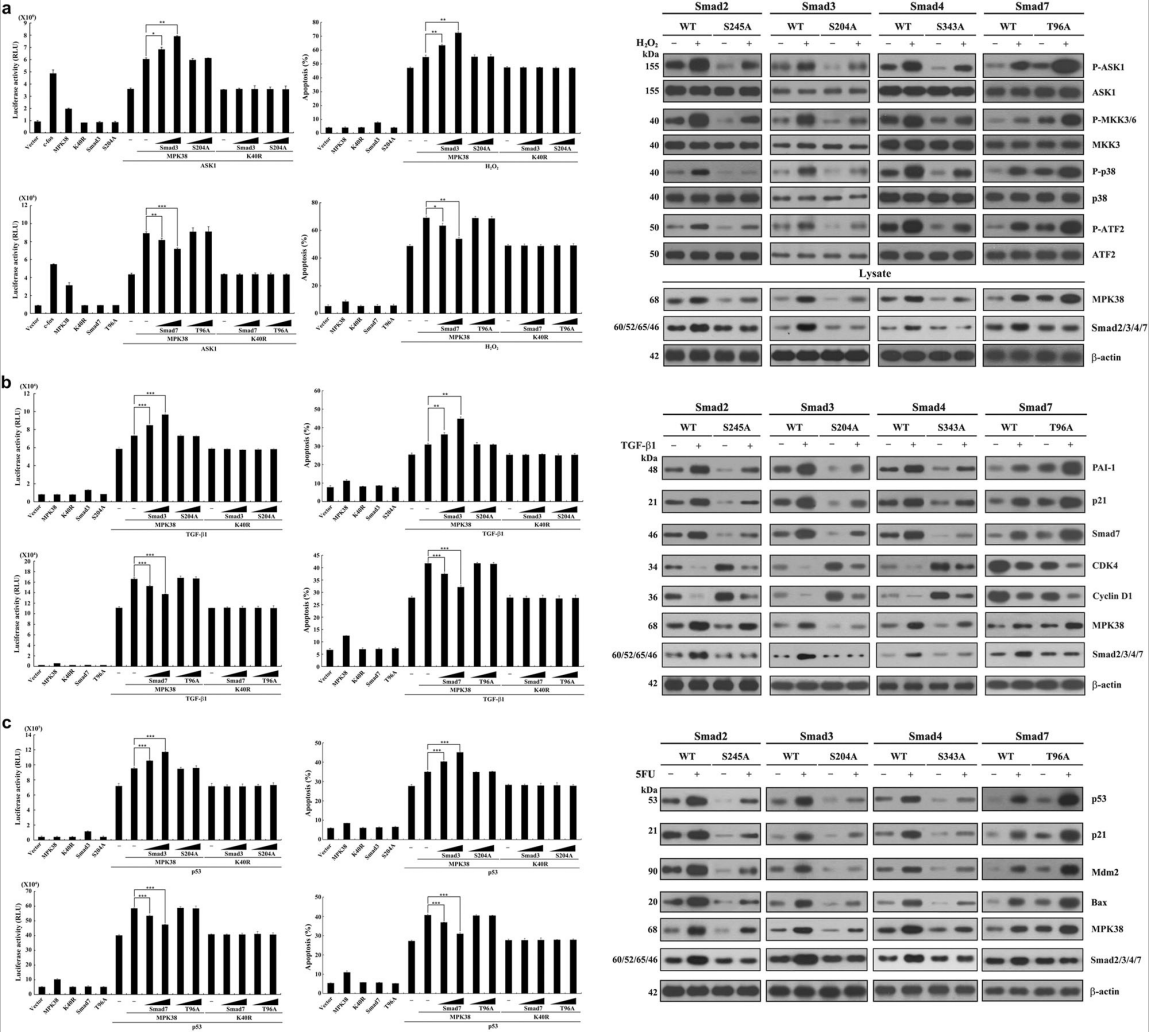
Differential regulation of MPK38‐mediated ASK1/TGF-β/p53 signaling by Smads. **a** 293T cells were transfected with various concentrations of vectors encoding WT or mutant Smads3/7 (0.5 and 1 μg), WT and K40R MPK38 (0.8 μg), WT ASK1 (0.6 μg), c‐fos (0.6 μg), or the AP‐1-luciferase plasmid (0.2 μg), as indicated. After 48 h, the cells were harvested, and luciferase activity was measured using a luciferase assay system (Promega). The total DNA concentration was kept constant by supplementation with empty vector DNA. The values were adjusted relative to the expression levels of a co-transfected β-galactosidase reporter control (left panels). HEK293 cells were transfected with various concentrations of vectors encoding WT and mutant Smads3/7 (0.6 and 1.2 μg), or WT and K40R MPK38 (0.8 μg), as indicated, in the presence or absence of H_2_O_2_ (1 mM, 9 h). Cells exposed only to H_2_O_2_ were used as a positive control. Apoptotic cell death was determined using a GFP system (middle panels). WT and CRISPR/Cas9 Smad knock-in HEK293 cells were treated with (+) or without (−) H_2_O_2_ (2 mM, 30 min), and the cell lysates were subjected to immunoprecipitation with antibodies for ASK1, MKK3, p38, and ATF2, followed by immunoblot analysis using anti‐phospho-specific antibodies for ASK1 Thr^845^ (Thr^838^ in human), MKK3/6 Ser^189/207^, p38 Thr^180^/Tyr^182^, and ATF2 Thr^71^. The protein levels of MPK38 and Smads in cell lysates were examined with anti-MPK38 and anti-Smads antibodies (right panels). *p < 0.05, **p < 0.01, ***p < 0.001 compared with MPK38 alone in the presence of ASK1 or H_2_O_2_. **b** HaCaT cells were transfected with various concentrations of vectors encoding WT or mutant Smads3/7 (0.5 and 1 μg), WT and K40R MPK38 (0.4 μg), or p3TP‐Lux plasmid (0.2 μg), as indicated, in the presence or absence of TGF‐β1 (100 pM) (left panels). HaCaT cells were transfected with various concentrations of vectors encoding WT or mutant Smads3/7 (0.5 and 1 μg) and/or WT and K40R MPK38 (0.4 μg), as indicated, together with an expression vector encoding GFP (1 μg). After treatment of the transfected cells with TGF‐β1 (2 ng/ml, 20 h), apoptotic cell death was then determined (middle panels). WT and CRISPR/Cas9 Smad knock-in HEK293 cells were treated with (+) or without (−) TGF‐β1 (2 ng/ml, 20 h), and the cell lysates were analyzed by immunoblot analysis using antibodies for PAI-1, p21, Smad7, CDK4, cyclin D1, MPK38, and Smads (Smad2, Smad3, Smad4, and Smad7) (right panels). **p < 0.01, ***p < 0.001 compared with MPK38 alone in the presence of TGF-β1. **c** MCF7 cells were transfected with various concentrations of vectors encoding WT or mutant Smads3/7 (0.5 and 1 μg), WT and K40R MPK38 (0.4 μg), or p53-Luc plasmid (0.2 μg), as indicated, in the presence or absence of p53 (0.3 μg) (left panels). MCF7 cells were transfected with various concentrations of vectors encoding WT or mutant Smads3/7 (0.5 and 1 μg) and/or WT and K40R MPK38 (0.4 μg), as indicated, together with an expression vector encoding GFP (1 μg) in the presence or absence of p53 (0.6 μg). Apoptotic cell death was then determined (middle panels). WT and CRISPR/Cas9 Smad knock-in HEK293 cells were treated with (+) or without (−) 5FU (0.38 mM, 30 h), and the cell lysates were analyzed by immunoblot analysis using antibodies for p53, p21, Mdm2, Bax, MPK38, and Smads (Smad2, Smad3, Smad4, and Smad7) (right panels). ***p < 0.001 compared with MPK38 alone in the presence of p53. The results represent the mean ± S.E. of at least three independent experiments performed in duplicate. Kinase-dead MPK38, K40R

Because MPK38 has been shown to potentiate coordinate ASK1/TGF-β/p53 signaling^7,11,23^, we first examined the effect of Smad proteins on ASK1/TGF-β/p53-mediated transcription induced by MPK38. As shown in Fig. 2 and Supplementary Fig. 1, the ASK1/TGF-β/p53-mediated transactivation induced by MPK38 increased in a kinase-dependent manner in the presence of wild-type Smads2/3/4, whereas MPK38-mediated phosphorylation-defective Smad mutants had no such effect. By contrast, Smad7 decreased the transcriptional activity. These results suggest that Smads2/3/4 are positive regulators of MPK38 and that Smad7 is a negative regulator of MPK38. The data also demonstrate that the phosphorylation of Smad proteins by MPK38 is critical for specific Smad-mediated regulation of ASK1/TGF-β/p53-mediated transactivation induced by MPK38.

We next examined whether Smad proteins can influence ASK1/TGF-β/p53-mediated apoptosis induced by MPK38. As shown in Fig. 2 (middle panels) and Supplementary Fig. 1, the coexpression of wild-type Smads2/3/4 and MPK38 resulted in a kinase-dependent increase in ASK1/TGF-β/p53-induced apoptosis compared with control cells transfected with MPK38 alone. However, the stimulatory effect of Smads2/3/4 on ASK1/TGF-β/p53-induced apoptosis was not observed in the presence of MPK38-mediated phosphorylation-defective Smad mutants. Conversely, the opposite effect was observed in the presence of Smad7. We also evaluated MPK38-mediated ASK1/TGF-β/p53 signaling activation using CRISPR/Cas9-mediated Smad KI HEK293 cells in the presence or absence of H_2_O_2_/TGF-β1/p53. As shown in Fig. 2 (right panels), and as expected, the activation of ASK1/TGF-β/p53 signaling was markedly lower in S245A, S204A, and S343A KI cells when compared with wild-type control cells, whereas T96A KI cells showed greater activation of ASK1/TGF-β/p53 signaling. Together, these findings suggest that MPK38-induced ASK1/TGF-β/p53 activity is positively regulated by Smads2/3/4, but negatively regulated by Smad7, in a MPK38 phosphorylation-dependent manner.

### MPK38 stability is increased by Smads2/3/4 but decreased by Smad7

**Fig. 3:**
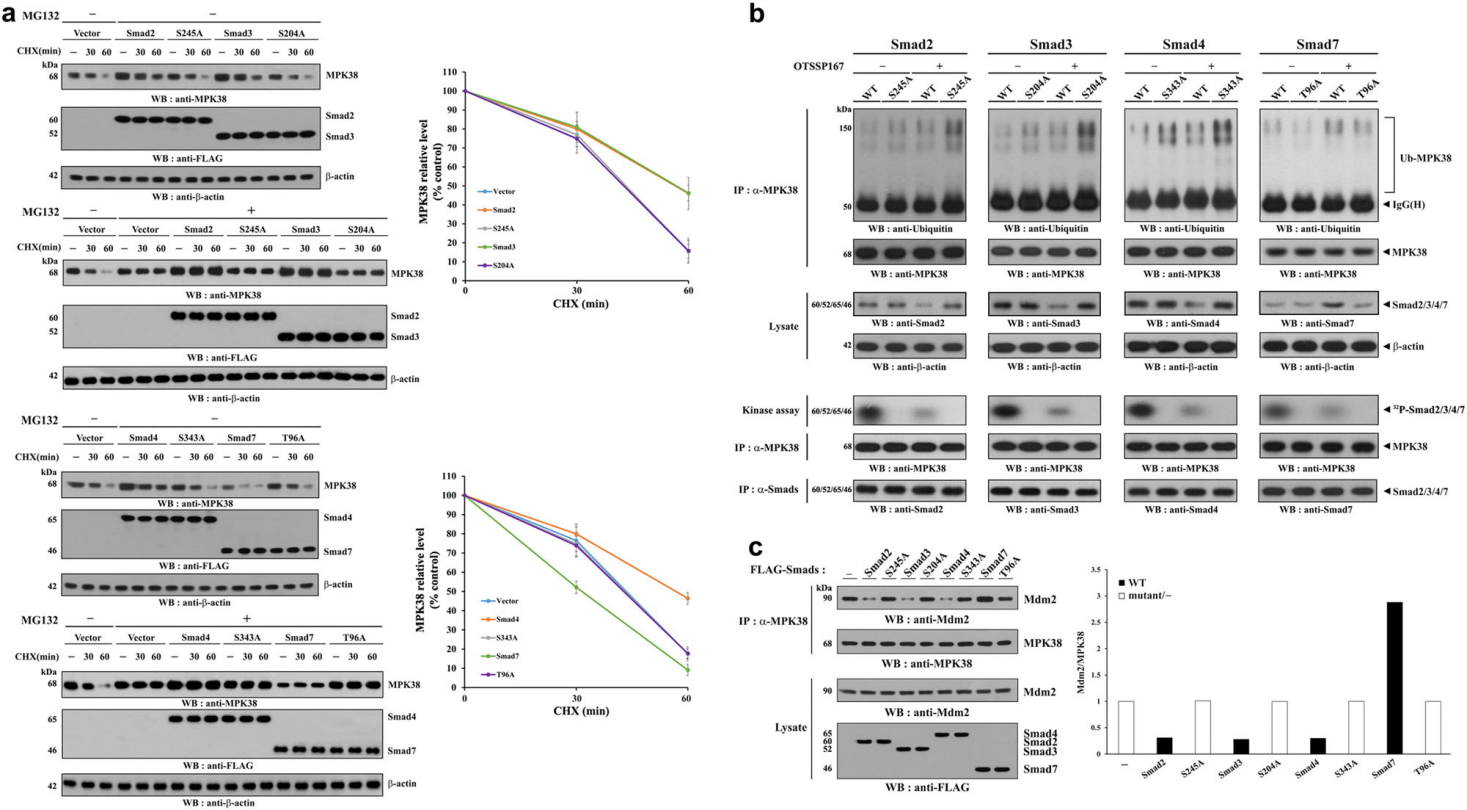
Differential regulation of MPK38 stability by Smads. **a** MPK38 protein stability was assessed by immunoblot analysis using an anti‐MPK38 antibody. HEK293 cells were transfected with pCMV2‐FLAG (vector) or vectors encoding FLAG-tagged WT or mutant Smads. Time intervals indicate the number of minutes after treatment with cycloheximide (CHX, 20 μg/ml) alone or with MG132 (10 μM). The MPK38 levels in at least three independent experiments were quantified by densitometry (right panels). The relative densitometry at each time point was expressed as a percentage of the density at time 0 min after normalization the corresponding β-actin level. **b** The ubiquitination of endogenous MPK38 was assessed using WT and CRISPR/Cas9 Smad knock-in HEK293 cells treated with (+) or without (−) OTSSP167 (1 μM, 2 h). **c** HEK293 cells were transfected with vectors encoding FLAG-tagged WT or mutant Smads, as indicated. Cell lysates were subjected to immunoprecipitation using an anti-MPK38 antibody (IP: α-MPK38) followed by immunoblot analysis using an anti‐Mdm2 antibody to determine the endogenous levels of MPK38‐Mdm2 complexes. Ratio of Mdm2/MPK38 was determined by density analysis of the bands, and the fold-increase relative to control not expressing Smads is presented

To address the mechanism of the differential regulation of MPK38 activity by Smads, we investigated the effect of Smads (Smad2, 3, 4, and 7) on MPK38 protein stability. HEK293 cells were transfected with expression vectors encoding wild-type and MPK38-mediated phosphorylation-defective Smad mutants, and MPK38 protein levels were quantified using immunoblot analysis. As shown in Fig. 3a, Smads2/3/4 increased the stability of MPK38 compared with control cells expressing empty vector, whereas Smad7 decreased MPK38 stability. However, such effects were not observed in the presence of MPK38-mediated phosphorylation-defective Smad mutants. Conversely, treatment of Smad-expressing HEK293 cells with both cycloheximide and MG132, a proteasomal inhibitor, led to greater stability of MPK38, compared with that in non-MG132-treated Smad-expressing HEK293 cells (Fig. 3a). These results indicate a critical role for the proteasome pathway in the effects of Smads on MPK38 degradation in cells. We then analyzed the effect of Smads on MPK38 ubiquitination using CRISPR/Cas9-mediated Smad KI HEK293 cells treated with (+) or without (−) OTSSP167. Smads2/3/4 endogenously decreased the ubiquitination of MPK38 in a MPK38 phosphorylation-dependent manner, whereas Smad7 increased MPK38 ubiquitination (Fig. 3b). These results suggest that, in addition to the class of Smad protein, MPK38-mediated phosphorylation of Smad proteins also plays a critical role in the regulation of MPK38 protein stability.

We then investigated whether the specific Smad-mediated regulation of MPK38 stability is dependent on the interaction between MPK38 and Mdm2^24^. HEK293 cells were transfected with expression vectors for wild-type and MPK38-mediated phosphorylation-defective Smad mutants. Smads2/3/4 markedly decreased MPK38-Mdm2 complex formation in a MPK38 phosphorylation-dependent manner, whereas Smad7 increased complex formation (Fig. 3c). These results also indicate the important role of MPK38-mediated phosphorylation of specific Smads in the differential regulation of Mdm2-dependent MPK38 ubiquitination. All these results suggest critical roles for specific Smad protein classes and their phosphorylation by MPK38 in the regulation of MPK38 stability: Smads2/3/4 stabilize MPK38, whereas Smad7 reduces its stability.

### Smad proteins differentially regulate complex formation between MPK38 and its regulators, thioredoxin (Trx) and zinc-finger-like protein 9 (ZPR9)

**Fig. 4:**
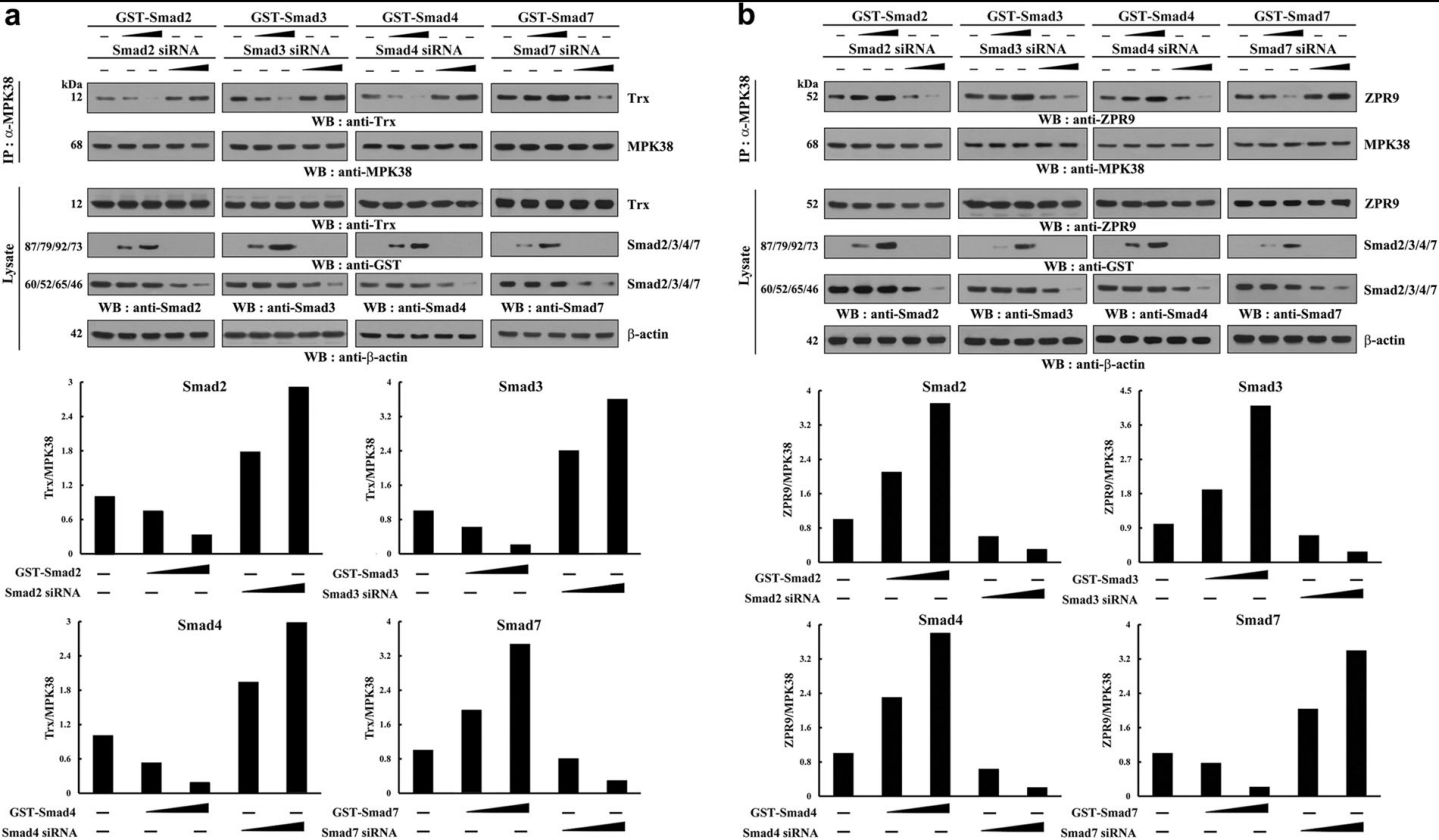
Smad-specific regulation of complex formation between MPK38 and Trx or ZPR9. HEK293 cells were transfected with various concentrations of vectors encoding GST-tagged Smads (0.5 and 1 μg) or Smad-specific siRNAs (100 and 200 nM), as indicated. Cell lysates were subjected to immunoprecipitation with an anti-MPK38 antibody (IP: α-MPK38), and endogenous complex formation between MPK38 and its regulators, Trx **a** and ZPR9 **b**, was assessed by immunoblot analysis using anti-Trx and anti-ZPR9 antibodies (upper panels). Ratios of Trx/MPK38 **a** and ZPR9/MPK38 **b** were determined by density analysis of the bands, and the fold-increase relative to controls not expressing Smads is presented (lower panels). Each experiment was repeated at least three times with similar results

To further explore the mechanism of differential regulation of MPK38 stability by Smads, we investigated whether Smad proteins affect the interaction between MPK38 and Trx, which destabilizes it^24^. HEK293 cells transfected with various quantities of plasmid vectors encoding Smads2/3/4/7 or specific Smad-targeting siRNAs were subjected to immunoprecipitation using an anti-MPK38 antibody, followed by immunoblot analysis using an anti-Trx antibody. There was a dose-dependent decrease in endogenous complex formation between MPK38 and Trx in cells expressing Smads2/3/4 compared with cells not expressing Smads. By contrast, Smad7 transfection increased complex formation (Fig. 4a). These results were corroborated by Smad silencing experiments using Smad-specific siRNAs (Fig. 4a). These findings indicate that Smad class type contributes to the Smad-mediated differential modulation of MPK38-Trx complex formation.

Because ZPR9 was recently shown to function as a stabilizer of MPK38^23^, we next investigated the effect of Smads on ZPR9 binding to MPK38. As expected, Smads2/3/4 increased endogenous complex formation between MPK38 and ZPR9, but Smad7 decreased this (Fig. 4b). Consistent with this, endogenous Smad silencing using Smad-specific siRNAs had the opposite effect on MPK38-ZPR9 complex formation (Fig. 4b). These observations provide evidence that the differential regulation of MPK38 stability by Smads is mediated by modulating complex formation between MPK38 and its regulators, Trx and ZPR9.

### Smads2/3/4 improve, but Smad7 worsens, glucose metabolism in a mouse model of diet-induced obesity

**Fig. 5:**
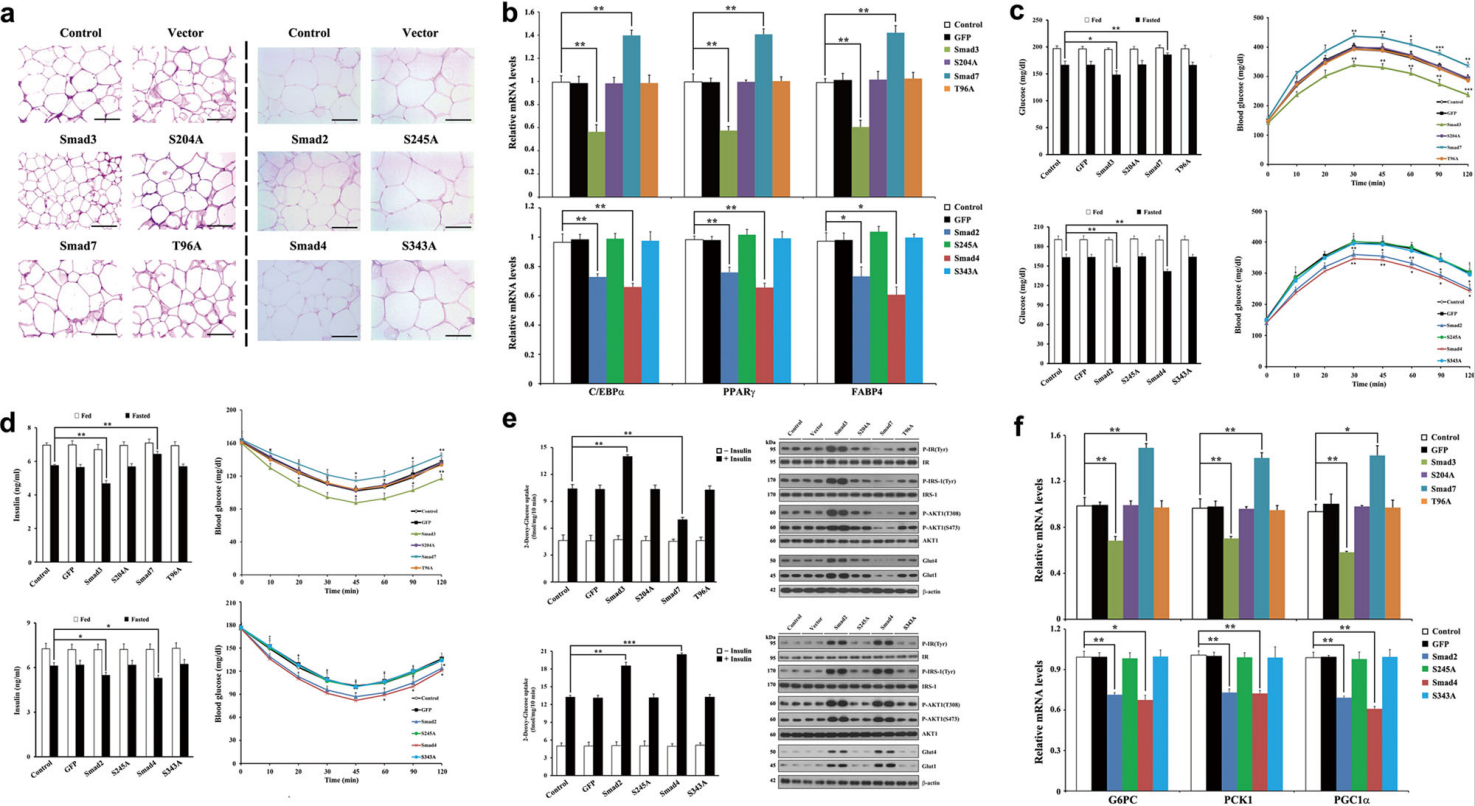
Specific effects of adenoviral delivery of Smads on glucose metabolism in diet-induced obese mice. **a** Hematoxylin and eosin (H&E)-stained paraffin-embedded sections of epididymal WAT in HFD-fed mice infected with the indicated adenoviruses. Scale bar, 100 μm. **b** Relative mRNA expression levels of adipogenic genes. mRNA expression was quantified by densitometry, and the fold-increase relative to control is presented. *n* = 6 per group, *p < 0.05, **p < 0.01 compared with control. **c**, **d** Blood glucose **c** and insulin **d** levels in fed and fasting (16 h) HFD-fed mice that had been infected with the indicated adenoviruses or not (control) (left panels). *n* = 6 per group, *p < 0.05, **p < 0.01 compared with the fasted controls, determined by two-way ANOVA. Glucose tolerance tests **c** and insulin tolerance tests **d** were conducted by measuring blood glucose concentrations in mice following intraperitoneal injection of glucose (2 g/kg) or insulin (0.75 U/kg), respectively. *n* = 6 per group, *p < 0.05, **p < 0.01, ***p < 0.001 compared with control, determined by two-way ANOVA. **e** In vitro ^3^H-2-deoxy-glucose uptake by epididymal WAT was measured in the presence or absence of human insulin (10 mU/ml) (left panels). *n* = 6 per group, **p < 0.01, ***p < 0.001 compared with the insulin-treated control, determined by two-way ANOVA. IRS-PI3K signaling was evaluated by immunoblot analysis (right panels) after in vivo insulin stimulation by injection into the inferior vena cava (*n* = 2 per group). **f** Relative mRNA expression levels of hepatic gluconeogenic genes. *n* = 6 per group, *p < 0.05, **p < 0.01 compared with control

Emerging evidence has implicated ASK1/TGF-β/p53 signaling pathways in the pathogenesis of obesity-associated metabolic diseases^8,25,26^. Our recent studies have shown that both genetically and diet-induced obese mice display lower levels of ASK1/TGF-β/p53 signaling and Smad3 expression, and a higher level of Smad7 expression, when compared with control wild-type or chow-fed mice^8,23^. Based on these findings, an adenoviral delivery system was employed to investigate whether Smad proteins regulate obesity-associated glucose metabolism by differentially regulating ASK1/TGF-β/p53 signaling in diet-induced obese mice. The adenoviral delivery of Smads2/3/4 significantly decreased cellular distribution toward extremely large, hypertrophic adipocytes, compared with uninfected HFD-fed mice or HFD-fed mice infected only with Green Fluorescent Protein (GFP)-expressing adenovirus (Ad-GFP) (Fig. 5a; Supplementary Fig. 2a). The mRNA expression levels of key adipogenic regulators, including CCAAT-enhancer-binding protein α, peroxisome proliferator-activated receptor γ (PPARγ), and fatty acid binding protein 4, were significantly lower in WAT from HFD-fed mice infected with Ad-Smads2/3/4 than those in the uninfected HFD-fed mice. However, Ad-Smad7 infection had an opposite effect (Fig. 5b).

Fasting blood glucose was lower in HFD-fed mice infected with Ad-Smads2/3/4 than in uninfected HFD-fed mice (Fig. 5c) and Ad-Smads2/3/4 infection enhanced glucose tolerance and insulin sensitivity in HFD-fed mice (Figs. 5c, d). In parallel, mice infected with Ad-Smads2/3/4 exhibited lower circulating insulin levels under fasting conditions, whereas Ad-Smad7 infection further increased the levels of circulating insulin (Fig. 5d, left). In addition, Ad-Smads2/3/4 infection significantly increased in vitro insulin-stimulated 2-deoxy-glucose uptake in WAT and muscle, whereas Ad-Smad7 infection had the opposite effect (Fig. 5e, left; Supplementary Fig. 2b). Consistent with this, we observed that Ad-Smads2/3/4 infection caused a significant upregulation of the insulin receptor substrate (IRS)-phosphoinositide 3-kinase (PI3K) pathway, which may contribute to enhanced glucose uptake, whereas the IRS-PI3K pathway was downregulated by Ad-Smad7 infection (Fig. 5e, right; Supplementary Fig. 1b). HFD-fed mice infected with Ad-Smads2/3/4 displayed a considerable decrease in blood glucose levels (Supplementary Fig. 2c), together with lower mRNA expression levels of hepatic gluconeogenic genes, including glucose-6-phosphatase (G6PC), phosphoenolpyruvate carboxykinase-1 (PCK1), and peroxisome proliferator-activated receptor γ coactivator 1α (PGC1α), when compared with uninfected HFD-fed mice (Fig. 5f). By contrast, no MPK38-mediated phosphorylation-defective Smad mutants had such an effect (Fig. 5; Supplementary Fig. 2). These results indicate that Smads2/3/4 improve, but Smad7 worsens, glucose metabolism in a MPK38 phosphorylation-dependent manner in diet-induced obese mice.

### Smads2/3/4 improve, but Smad7 worsens, lipid metabolism and inflammation in a mouse model of diet-induced obesity

**Fig. 6:**
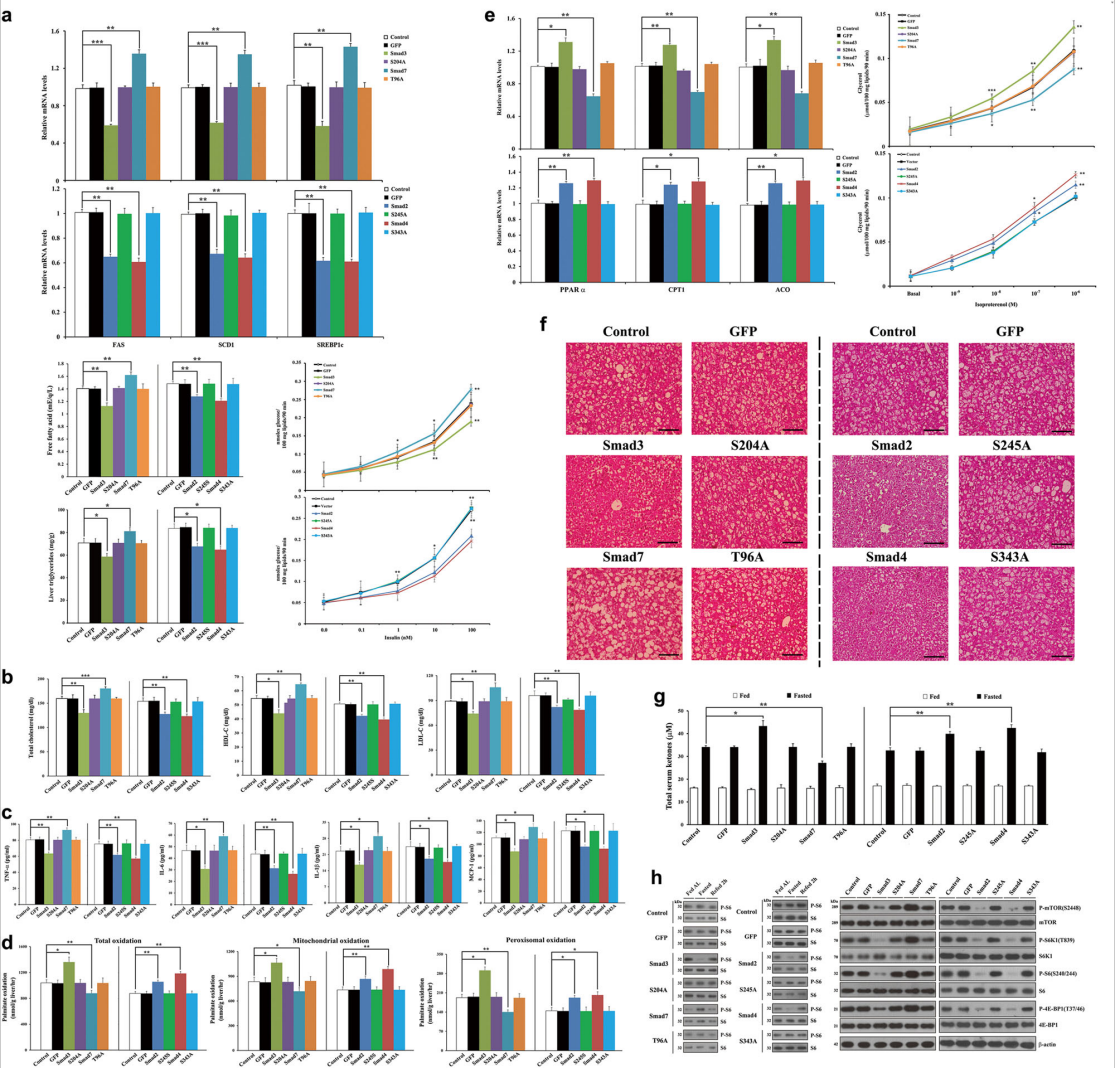
Specific regulation of lipid metabolism and inflammation by adenoviral delivery of Smads to diet-induced obese mice. **a**-**c** Relative mRNA expression levels of lipogenic genes in epididymal WAT, and blood free fatty acid concentration **a**, lipogenic capacity of hepatocytes **a**, liver triglyceride concentration **a**, circulating total cholesterol, HDL-C, and LDL-C concentrations **b**, and serum concentrations of proinflammatory proteins **c**. *n* = 6 per group, *p *<* 0.05, **p < 0.01, ***p < 0.001 compared with control. **d** Measurement of β-oxidation using ^14^C-labeled palmitate in liver. *n* = 6 per group, *p < 0.05, **p < 0.01 compared with control. **e** Relative mRNA expression levels of fatty acid oxidative genes in epididymal WAT (left) and the isoproterenol-stimulated lipolytic response in isolated adipocytes (right). *n* = 6 per group, *p < 0.05, **p < 0.01, ***p < 0.001 compared with control. **f** Representative images of H&E-stained sections of livers. *n* = 6 per group. Scale bar, 100 μm. **g** Measurement of total ketone bodies in fed and fasted (24 h) blood. *n* = 6 per group, *p < 0.05, **p < 0.01 compared with fasted controls, determined by two-way ANOVA. **h** Phospho-S6 Ser^240/244^ levels in liver lysates from ad libitum-fed, fasted (24 h), and re-fed (2 h) HFD-fed mice infected or not (control) with the indicated adenoviruses (left panels). Immunoblot analyses of the mTORC1 signaling pathway using liver lysates (right panels). *n* = 6 per group

We then examined whether Smad proteins regulate lipogenic gene expression in HFD-fed mice. Ad-Smads2/3/4 infection significantly reduced the mRNA expression of adipose and hepatic lipogenic genes, including fatty acid synthase (FAS), sterol CoA desaturase 1, and sterol regulatory element-binding transcription factor 1c, consistent with lower circulating free fatty acid levels, whereas Ad-Smad7 infection had the opposite effect in both tissues (Fig. 6a; Supplementary Fig. 3a). Consistent with this, and in contrast to the effects of infection with Ad-Smad7, HFD-fed mice infected with Ad-Smads2/3/4 exhibited lower lipogenesis (Fig. 6a), liver triglyceride (Fig. 6a), circulating total cholesterol, high-density lipoprotein (HDL)-cholesterol, and low-density lipoprotein (LDL)-cholesterol (Fig. 6b). However, there was no such effect in the presence of MPK38-mediated phosphorylation-defective Smad mutants. These results suggest that Smad phosphorylation by MPK38 is important in the Smad-mediated differential regulation of lipogenesis. A previous report demonstrated that cholesterol, fatty acids, and modified lipids activate inflammatory pathways and modulate the activity of leukocytes^27^. Therefore, we also examined the expression of proinflammatory genes in blood samples. Ad-Smads2/3/4 infection considerably decreased serum proinflammatory proteins when compared with uninfected HFD-fed mice, whereas Ad-Smad7 infection increased the serum levels of proinflammatory proteins (Fig. 6c). However, these effects were not observed after infection with MPK38-mediated phosphorylation-defective Smad mutants. These results indicate that Smads2/3/4 have beneficial effects, whereas Smad7 has an adverse effect, on lipogenesis and inflammation.

Ad-Smads2/3/4 infections also increased the mRNA expression of adipose lipolytic genes, including hormone-sensitive lipase (HSL), adipose triglyceride lipase (ATGL), and beta-3 adrenergic receptor (ADRB3), in a MPK38 phosphorylation-dependent manner, whereas Ad-Smad7 infection decreased the expression of these genes (Supplementary Fig. 3b). Consistent with this finding, Ad-Smads2/3/4 infection stimulated hepatic fatty acid utilization by stimulating the oxidation of fatty acids via mitochondrial and peroxisomal β-oxidation pathways in a MPK38 phosphorylation-dependent manner, whereas Smad7 had the opposite effect on fatty acid utilization (Fig. 6d). Indeed, WAT from HFD-fed mice infected with Ad-Smads2/3/4 exhibited higher expression of key genes involved in fatty acid oxidation, such as peroxisome proliferator-activated receptor α (PPARα), carnitine palmitoyltransferase 1, and acyl-CoA oxidase, as well as lower levels of blood triglycerides and higher isoproterenol-stimulated lipolysis (Fig. 6e; Supplementary Fig. 3c). HFD-fed mice infected with Ad-Smads2/3/4 also displayed substantial decreases in liver lipid accumulation and triglyceride content (Fig. 6a, f). However, we observed the opposite trend in HFD-fed mice infected with Ad-Smad7. All these results indicate the positive roles of Smads2/3/4 and the negative role of Smad7 in lipid oxidation.

Because abnormal mitochondrial fat oxidation is associated with insulin resistance and impaired ketogenesis^28^, we also investigated whether Smad proteins differentially regulate hepatic ketogenesis. As expected, Ad-Smads2/3/4 infection enhanced ketone body production (Fig. 6g) and ketogenic gene expression (Supplementary Fig. 3d) under fasting conditions when compared with uninfected HFD-fed mice, but Ad-Smad7 infection had the opposite effect. We also analyzed mechanistic target of rapamycin complex 1 (mTORC1) signaling in HFD-fed mice infected with the indicated adenoviruses because mTORC1 signaling has previously been shown to be reciprocally regulated with respect to ketogenesis^29^. In contrast to the effects of Ad-Smad7 infection, liver lysates from HFD-fed mice infected with Ad-Smads2/3/4 displayed lower levels of phospho-S6 Ser^240/244^ in response to fasting compared with the levels in uninfected HFD-fed mice (Fig. 6h, left panels). Immunoblot analyses of the mTORC1 signaling pathway also confirmed that Smads2/3/4 and Smad7 are responsible for downregulating and upregulating mTORC1 signaling, respectively (Fig. 6h, right panels). These results suggest that Smads2/3/4 can significantly improve the hyperlipidemic phenotype in HFD-fed mice by upregulating MPK38-dependent ASK1/TGF-β/p53 signaling (Supplementary Fig. 4) and downregulating mTORC1 signaling (Fig. 6h), and that Smad7 worsens the hyperlipidemic state in HFD-fed mice by exerting opposing effects.

## Discussion

Growing evidence underscores the importance of Smads in obesity-associated metabolism, although Smad proteins are best known for their roles as transcription factors in the TGF‐β signaling pathway. However, the mechanisms underlying the regulation of cellular metabolism by Smads remain poorly understood. We thus investigated the role of Smads in the regulation of the activity of the protein kinase MPK38, which plays a critical role in the coordinate activation of ASK1/TGF‐β/p53 signaling that is closely associated with metabolic homeostasis^25,26,30,31^, and found that Smad proteins differentially regulate MPK38 function through direct interactions, suggesting that Smad proteins, in addition to their roles as transcription factors, may function as regulators of MPK38. To investigate the mechanism whereby MPK38 activity is regulated by Smads, we examined whether the MPK38-mediated phosphorylation of Smads could affect the activity of MPK38, using various biochemical analyses (Figs. 1 and 2). Smads2/3/4 markedly stimulate ASK1/TGF-β/p53 signaling, whereas Smad7 inhibits this. However, such effects were not observed in the presence of MPK38-mediated phosphorylation-defective Smad mutants. These findings indicate that Smad proteins have specific regulatory effects on MPK38 activity, depending on their class, but also that these effects are strictly dependent on Smad phosphorylation by MPK38. All these results strongly suggest that Smad proteins have novel roles in the differential regulation of cellular protein kinases. In addition to the effects on MPK38, we also observed a similar trend in the effect of Smads on ASK1 activity^8^.

To further enhance understanding of the mechanism(s) by which Smad proteins differentially regulate MPK38 activity, we also found that they modify the stability and ubiquitination of MPK38. However, such effects were not detected in MPK38-mediated phosphorylation-defective Smad mutants, indicating that Smad phosphorylation by MPK38 is required for these regulatory effects (Fig. 3). These results suggest that Smad proteins differentially regulate complex formation between MPK38 and its known regulators, Trx and ZPR9, through direct interactions with MPK38, because Trx and ZPR9 destabilize and stabilize MPK38 through Thr^76^ and Thr^252^ phosphorylation, respectively^23,24^. Indeed, the present study demonstrates that Smads2/3/4 decrease MPK38-Trx complex formation and increase MPK38-ZPR9 complex formation, whereas Smad7 has the opposite effect (Fig. 4). These observations support a model in which Smad proteins differentially modify MPK38-Trx and/or MPK38-ZPR9 complex formation depending on their class and contribute to the differential regulation of MPK38 kinase activity, resulting in specific regulation of MPK38-dependent ASK1/TGF‐β/p53 signaling pathways (Supplementary Fig. 5).

Obese mice display lower MPK38 kinase activity^23^, ASK1/TGF-β/p53 signaling^8^, and Smads2/3/4 expression, and higher levels of Smad7 expression (Supplementary Fig. 6), compared with control mice. These results raise the possibility that Smad proteins are involved in obesity-associated metabolism, probably by regulating the activation of ASK1/TGF‐β/p53 signaling through MPK38. To test this hypothesis, we employed an adenoviral delivery system to restore the lower levels of ASK1/TGF-β/p53 signaling in obese mice and analyzed the effect of Smads on obesity-associated metabolic abnormalities in HFD-fed mice. Forced expression of Smads2/3/4, but not MPK38-mediated phosphorylation-defective Smad mutants, induced a significant increase in ASK1/TGF-β/p53 signaling activation (Supplementary Fig. 4) and ameliorated glucose and lipid metabolism (Figs. 5 and 6) in HFD-fed mice versus uninfected mice fed a HFD, whereas Ad-Smad7 infection decreased ASK1/TGF-β/p53 signaling and further worsened the impaired glucose and lipid metabolism in a MPK38 phosphorylation-dependent manner. In addition, MPK38 kinase activity was increased by Smads2/3/4 but decreased by Smad7 in HFD-fed mice (Supplementary Fig. 4).

In conclusion, our data show that Smad proteins differentially regulate glucose and lipid metabolism and inflammation in diet-induced obese mice by differentially regulating MPK38‐dependent ASK1/TGF-β/p53 signaling, and that this effect is dependent on MPK38-mediated Smad phosphorylation and Smad class. Smads2/3/4 function as positive regulators of MPK38, whereas Smad7 functions as a negative regulator of MPK38. Moreover, this novel function of Smads as differential regulators of MPK38‐dependent ASK1/TGF‐β/p53 signaling provides important information about the mechanism determining how each class of Smad protein contributes to the maintenance of metabolic homeostasis in mice.

## Materials and methods

### Antibodies, plasmids, chemicals, cell culture, and isolation of hepatocytes and adipocytes

Antibodies and plasmids used for experiments have been described previously^7,8,23^. Cycloheximide (CHX) was from Sigma-Aldrich. All other chemicals used was described^8,23^. Cell culture and isolation of hepatocytes and adipocytes were also described^8,23^.

### Generation of Smad KI cell lines

Genomic mutations were generated in HEK293 cells using the CRISPR/Cas9 system, as described previously^23^. Briefly, single-guide (sg) RNAs were designed to target the genomic areas adjacent to the Smad mutation sites (Supplementary Table 1). Two complementary oligonucleotides (Supplementary Table 2) containing the appropriate Smad guide sequence and Bbs1 ligation adapters were synthesized by Bioneer Ltd. (Cheongwon, Korea). The annealed oligonucleotides were ligated into a Bbs1-digested pX458 vector (Addgene plasmid no. 48138) using the Quick-Ligation system (New England BioLabs). To generate the Smad KI cell lines, HEK293 cells were cultured on a 24-well plate to ~60% confluence and co-transfected with 1 μg Smad sg RNA plasmid and pUC19 Smad (Smad2 S245A, Smad3 S204A, Smad4 S343A, or Smad7 T96A) using Lipofectamine 2000 (Invitrogen). After culturing in a 96-well plate, GFP-positive cells were identified, followed by genomic DNA extraction. Smads were amplified by PCR using Smad-specific PCR primer pairs (Supplementary Table 3). The PCR products were A-tailed and cloned into the pGEM-T Easy vector (Promega) to confirm the identity of individual Smad KI clones by DNA sequencing. The in vivo phosphorylation of Smads by MPK38 was validated using in vitro kinase assays (Supplementary Fig. 7).

### Coimmunoprecipitation, immunoblotting, MPK38 kinase assay, apoptosis assay, RNA isolation, and quantitative PCR (qPCR)

Coimmunoprecipitation, immunoblot analysis, and MPK38 kinase assay were performed as previously described using Smad KI cells or HEK293 cells transiently transfected with the indicated expression vectors or Smad-specific siRNAs^7,11^. Apoptosis and qPCR were performed as described previously^8,11^.

### Glucose and insulin tolerance tests (GTTs/ITTs), lipogenesis/lipolysis assays, and blood metabolic parameters

Plasma glucose levels for GTTs/ITTs were measured using blood obtained from the tail vein at 0, 10, 20, 30, 45, 60, 90, and 120 min post-injection according to the Mouse Metabolic Phenotyping Center recommendations for data presentation^32^. Lipogenesis and lipolysis were assayed in hepatocytes and adipocytes obtained from HFD-fed mice infected with the indicated adenoviruses, as described previously^8^. Serum was obtained by centrifugation of blood samples collected from the abdominal aorta of 12–14-week-old HFD-fed mice and stored at −70 °C. Serum levels of tumor necrosis factor (TNF)-α, IL-1β, IL-6, and monocyte chemoattractant protein 1 (MCP1) were determined using analysis kits from Peprotech (ADI-900-047), RayBio (ELM-IL1beta-001), Invitrogen (KMC0061), and R&D Systems (MJE00), respectively. Other blood metabolic parameters were measured as described previously^8^.

### Serum insulin and glucose, liver triglyceride, 2-deoxy-glucose uptake, total ketone, and fatty acid β-oxidation

Serum insulin and glucose levels, liver triglyceride content, and 2-deoxy-glucose uptake in epididymal WAT and soleus muscles were measured as described previously^8,33^. Total ketone levels were measured using a colorimetric assay from Wako Chemicals^8^. Fatty acid oxidation rate was determined using [1-^14^C] palmitic acid, as described previously^8^.

### Animal experiments and adenoviral infection

Four-week-old male C57BL/6 mice purchased from Orient (Seongnam, Korea) were fed a HFD (60% kcal as fat diet, D12492; Research Diets, Inc.) for 8–10 weeks under a 12:12-h light:dark cycle. Male 12- to 14-week-old C57BL/6 mice fed with a standard rodent diet were used as controls. All procedures were performed in a specific pathogen-free facility at Chungbuk National University, in accordance with the approved animal protocols and the guidelines established by the Ethics Review Committee of Chungbuk National University for Animal Experiments (CBNUA-966-16-02). Recombinant adenoviruses were generated as previously described^34^. To prepare adenoviruses expressing wild-type and MPK38 phosphorylation-defective Smad mutants, FLAG-tagged wild-type and mutant Smad plasmids were used as templates for PCR with the primers shown in Supplementary Table 4. Recombinant adenoviruses (approximately 1 × 10^9^ plaque-forming units) were directly injected into the tail vein or epididymal fat pads of 12- to 14-week-old HFD-fed mice.

### Data analysis

Data are expressed as mean ± standard error and are representative of at least three independent experiments. Statistical significance was determined by one-way or two-way analysis of variance (ANOVA), followed by Tukey’s multiple comparison test, using GraphPad Prism software (GraphPad Software).

## Electronic supplementary material

Supplemental Information
