# Supplementary material for: Smad proteins differentially regulate obesity-induced glucose and lipid abnormalities and inflammation via class-specific control of AMPK-related kinase MPK38/MELK activity
Source: Cell Death Dis. 2018 Apr 27;9(5):471. doi: 10.1038/s41419-018-0489-x (PMC5920110; doi:10.1038/s41419-018-0489-x)
Supplement: Supplementary file 1 — Supplemental Information [file 41419_2018_489_MOESM1_ESM.pdf]

Supplemental Information

Smad proteins differentially regulate obesity-induced glucose and lipid abnormalities and inflammation via class-specific control of AMPK-related kinase MPK38/MELK activity

Running Title: Smad-specific regulation of MPK38/MELK activity

Hyun-A Seong[1], Ravi Manoharan[2], and Hyunjung Ha[1,*]

[1]Department of Biochemistry, School of Biological Sciences, Chungbuk National University, Cheongju 28644, Republic of Korea

[2]Department of Biochemistry, University of Madras, Guindy Campus, Chennai 600025, India

Address correspondence to:

Hyunjung Ha

Department of Biochemistry, School of Biological Sciences, Chungbuk National University, Cheongju 28644, Republic of Korea. Tel.: +82-43-261-3233; Fax: +82-43-267-2306; E-mail: hyunha@cbnu.ac.kr

Supplementary Figure Legends

Supplementary Fig. 1 Differential regulation of MPK38-dependent ASK1/TGF-β/p53-mediated transcription and apoptosis by Smad2/4.

a Differential regulation of MPK38-dependent ASK1-mediated transcription and apoptosis by Smad2/4. To assess the effects of Smad2/4 on ASK1-mediated transcription, 293T cells were transfected with various concentrations of vectors encoding WT or mutant Smads2/4 (0.5 and 1 μg), WT and K40R MPK38 (0.8 μg), WT ASK1 (0.6 μg), c-fos (0.6 μg), or the AP-1-luciferase plasmid (0.2 μg), as indicated. To assess the effects of Smad2/4 on H_2O_2-mediated apoptosis, HEK293 cells were transfected with various concentrations of vectors encoding WT and mutant Smads2/4 (0.6 and 1.2 μg), or WT and K40R MPK38 (0.8 μg), as indicated, in the presence or absence of H_2O_2 (1 mM, 9 h). $^{**}p < 0.01$, $^{***}p < 0.001$ compared with MPK38 alone in the presence of ASK1 or H_2O_2. **b** Differential regulation of MPK38-dependent TGF-β-mediated transcription and apoptosis by Smad2/4. To assess the effects of Smad2/4 on TGF-β-mediated transcription, HaCaT cells were transfected with various concentrations of vectors encoding WT or mutant Smads2/4 (0.5 and 1 μg), WT and K40R MPK38 (0.4 μg), or p3TP-Lux plasmid (0.2 μg), as indicated, in the presence or absence of TGF-β1 (100 pM). To assess the effects of Smad2/4 on TGF-β-mediated apoptosis, HaCaT cells were transfected with various concentrations of vectors encoding WT or mutant Smads2/4 (0.5 and 1 μg) and/or WT and K40R MPK38 (0.4 μg), as indicated, together with an expression vector encoding GFP (1 μg). After treatment of the transfected cells with TGF-β1 (2 ng/ml, 20 h), GFP-based apoptotic cell death was determined (right panels). $^{***}p < 0.001$ compared with MPK38 alone in the presence of TGF-β1. **c** Differential regulation of MPK38-dependent p53-mediated transcription and apoptosis by Smad2/4. To assess the effects of Smad2/4 on p53-mediated transcription, MCF7 cells were transfected with various concentrations of vectors encoding WT or mutant Smads2/4 (0.5 and 1 μg), WT and K40R MPK38 (0.4 μg), or p53-Luc plasmid (0.2 μg), as indicated, in the presence or absence of p53 (0.3 μg). To assess the

effects of Smad2/4 on p53-mediated apoptosis, MCF7 cells were transfected with various concentrations of vectors encoding WT or mutant Smads2/4 (0.5 and 1 μg) and/or WT and K40R MPK38 (0.4 μg), as indicated, together with an expression vector encoding GFP (1 μg) in the presence or absence of p53 (0.6 μg). Apoptotic cell death was then determined. ***$p <$ 0.001 compared with MPK38 alone in the presence of p53. The expression levels of HA-ASK1, GST-MPK38, FLAG-p53 and FLAG-Smad2/3/4/7 were determined by immunoblotting with anti-HA, anti-GST, and anti-FLAG antibodies. Kinase-dead MPK38, K40R.

Supplementary Fig. 2 Smad-specific regulation of glucose metabolism in HFD-fed mice. **a** Size distribution analysis of adipocytes was performed in HFD-fed mice infected with the indicated adenoviruses, as described previously[8]. n = 6 per group. **b** *In vitro* ^{3}H-2-deoxy-glucose uptake by soleus muscles was measured in the presence or absence of human insulin (10 mU/ml) (left panels). n = 6 per group, *$p < 0.05$, **$p < 0.01$, ***$p < 0.001$ compared with control treated with insulin, determined by two-way ANOVA. IRS-PI3K signaling was evaluated by immunoblot analyses (right panels) after *in vivo* insulin stimulation by injection into the inferior vena cava (n = 2 per group). **c** Serum level of glucose, determined using an automated serum analyzer. n = 6 per group, *$p < 0.05$, **$p < 0.01$ compared with control.

Supplementary Fig. 3 Smad-specific regulation of lipid metabolism in HFD-fed mice.

a Relative mRNA expression levels of lipogenic genes in liver. n = 6 per group, *$p < 0.05$, **$p < 0.01$, ***$p < 0.001$ compared with control. **b** Relative mRNA expression levels of lipolytic genes in epididymal WAT. n = 6 per group, *$p < 0.05$, **$p < 0.01$, ***$p < 0.001$ compared with control. **c** Serum triglycerides, determined by an automated serum analyzer. n = 6 per group, *$p < 0.05$, **$p < 0.01$ compared with control. **d** Relative mRNA levels of genes involved in ketogenesis, including PPARα, CPT1, and 3-hydroxy-3-methylglutaryl-CoA synthase 2

(HMGCS2), in livers. Phosphoenolpyruvate carboxykinase (PEPCK), which is not a target of PPARα, was used as a nonspecific control. n = 6 per group, ***p* < 0.01, ****p* < 0.001 compared with fasted control, determined by two-way ANOVA.

Supplementary Fig. 4 Smad-specific regulation of ASK1/TGF-β/p53 signaling and MPK38 kinase activity in HFD-fed mice.

ASK1/TGF-β/p53 signaling and the kinase activity of MPK38 in hepatocytes and adipocytes derived from HFD-fed mice infected with the indicated adenoviruses were analyzed by immunoblot analysis using the indicated antibodies and the anti-phospho-specific antibodies described in Fig. 1a, respectively. The expression level of MPK38 in cell lysates was examined with an anti-MPK38 antibody. The relative levels of phosphorylation and expression were quantified by densitometry, and the fold increase relative to control is presented. Control, uninfected HFD-fed mice. The experiments were repeated at least three times with similar results.

Supplementary Fig. 5 Schematic diagram of the roles of Smads in the regulation of MPK38-dependent ASK1/TGF-β/p53 signaling pathways.

Obese mice display lower Smads2/3/4 expression and higher levels of Smad7 expression compared to control lean mice, leading to the downregulation of ASK1/TGF-β/p53 signaling through differential regulation of MPK38 kinase activity.

Supplementary Fig. 6 Comparison of the expression levels of Smads2/3/4/7 and the activation levels of p53 signaling between mice fed a control chow diet and a HFD.

Primary hepatocytes derived from Chow- and HFD-fed C57BL/6 mice (**a**) and isolated

hepatocytes treated (+) or untreated (-) with 5FU (0.38 mM, 30 h) (**b**) were subjected to immunoblot analysis using the indicated antibodies to examine the protein expression levels. The experiments were repeated at least three times with similar results.

Supplementary Fig. 7 Demonstration of *in vivo* phosphorylation of Smads2/3/4/7 by MPK38 in CRISPR/Cas9 Smad knock-in cells.

a Immunoprecipitated MPK38 (upper panels) and recombinant MPK38 (lower panels) were assayed for their kinase activity in the presence of Smad2/3/4/7 immunoprecipitates obtained from cell lysates of WT and clonal CRISPR/Cas9 Smad knock-in isolates as substrates. **b** In vitro kinase assays were carried out with immunoprecipitated Smad2/3/4/7 as substrates in the absence of MPK38. Each number represents an individual clone number of CRISPR/Cas9 Smad KI isolates. WT, wild-type; Cons., control; KI, knock-in.

Supplementary Fig.1

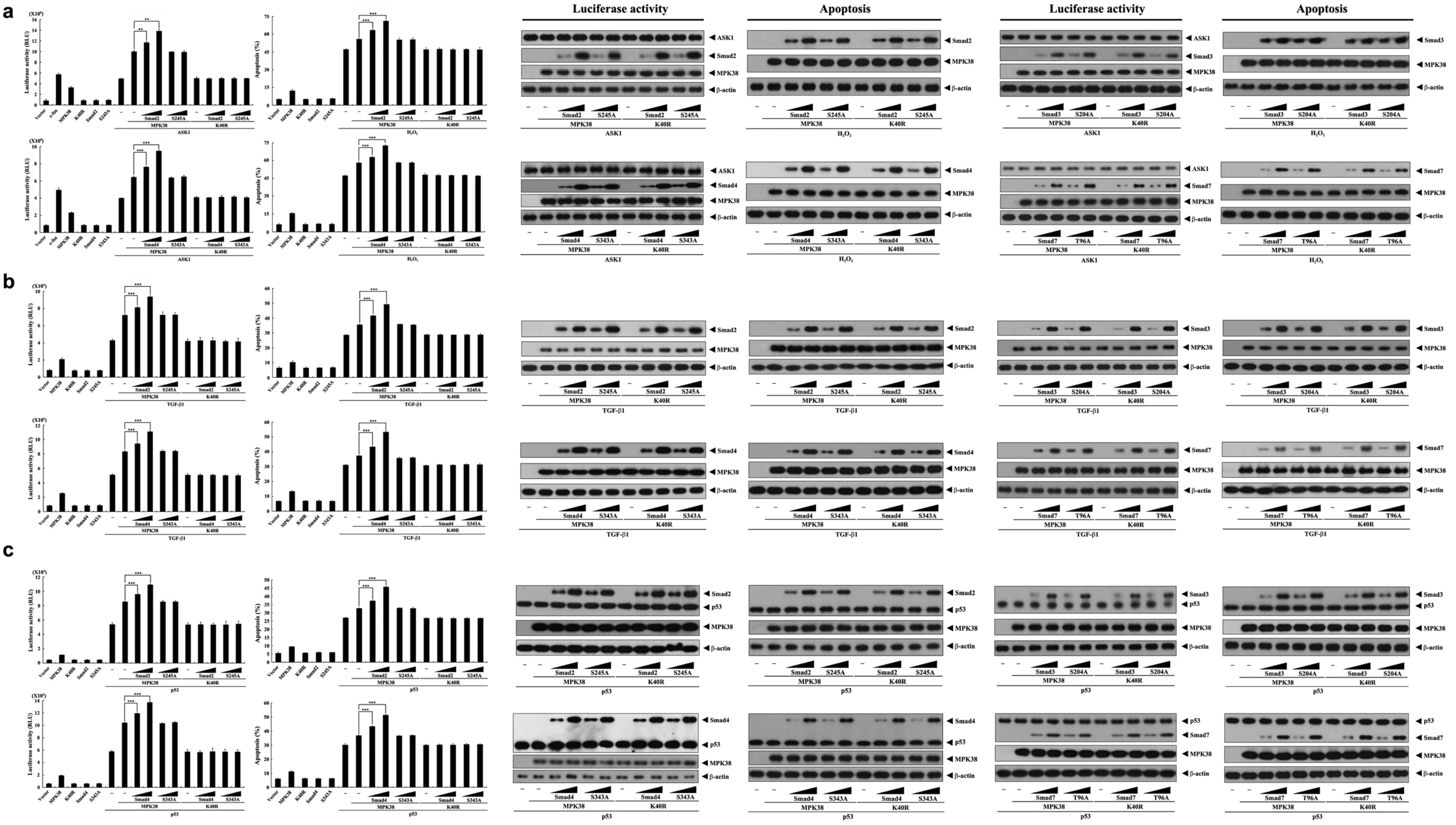

Supplementary Fig. 2

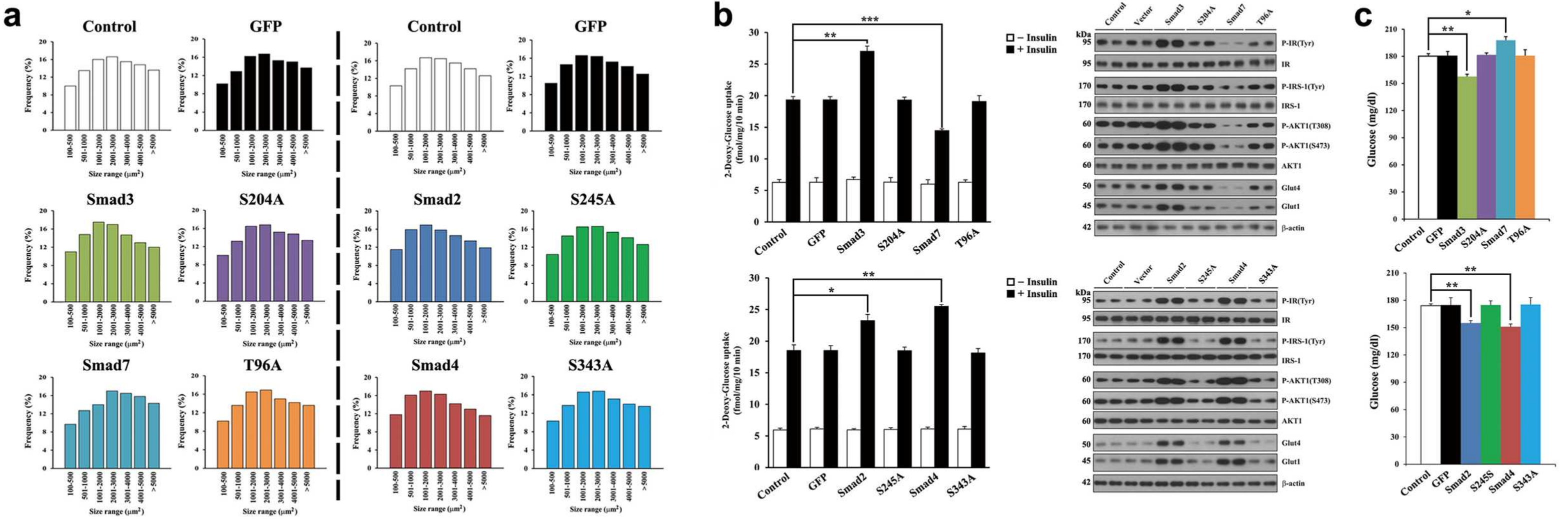

Supplementary Fig. 3

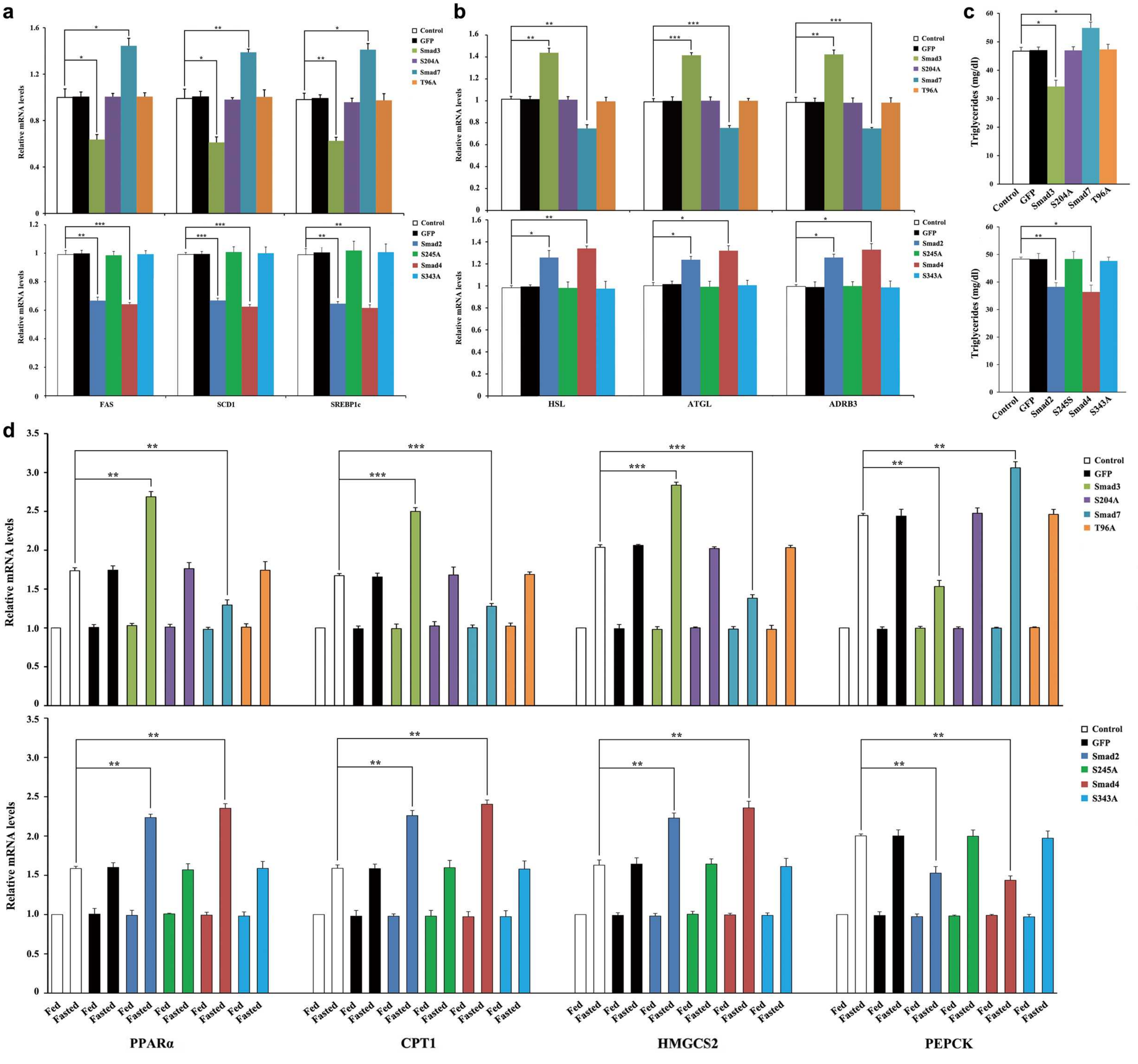

Supplementary Fig. 4

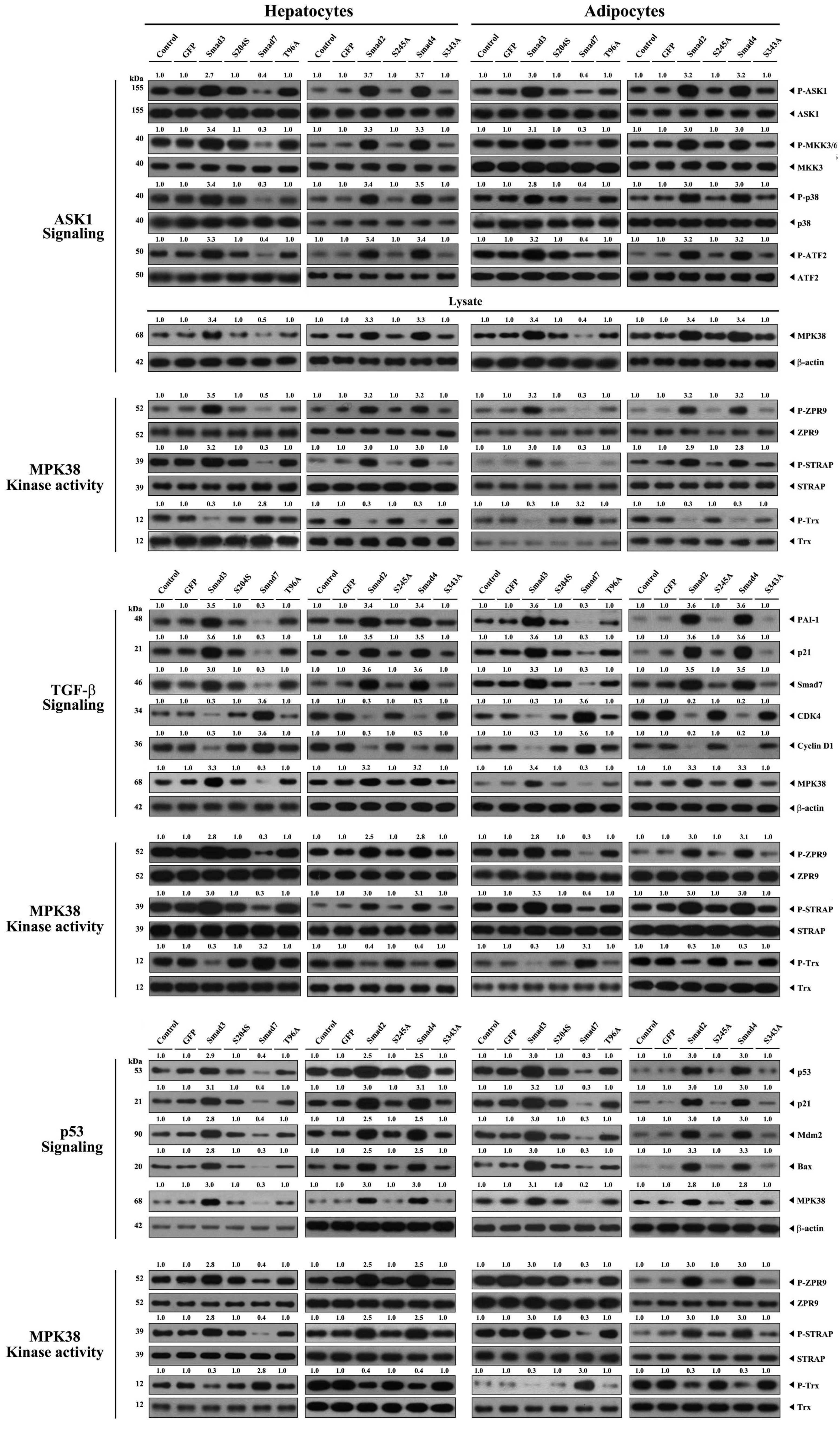

Supplementary Fig.5

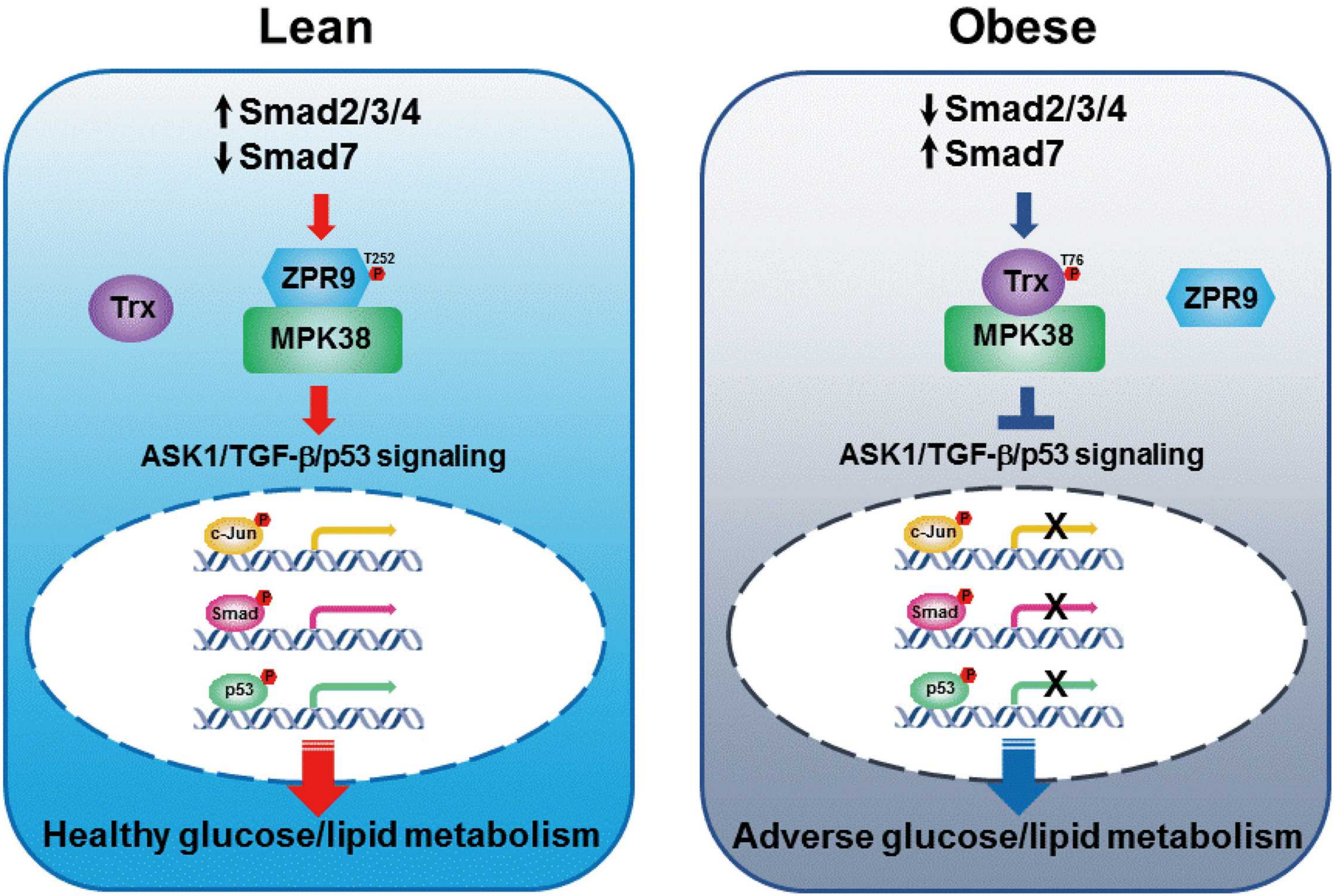

Supplementary Fig.6

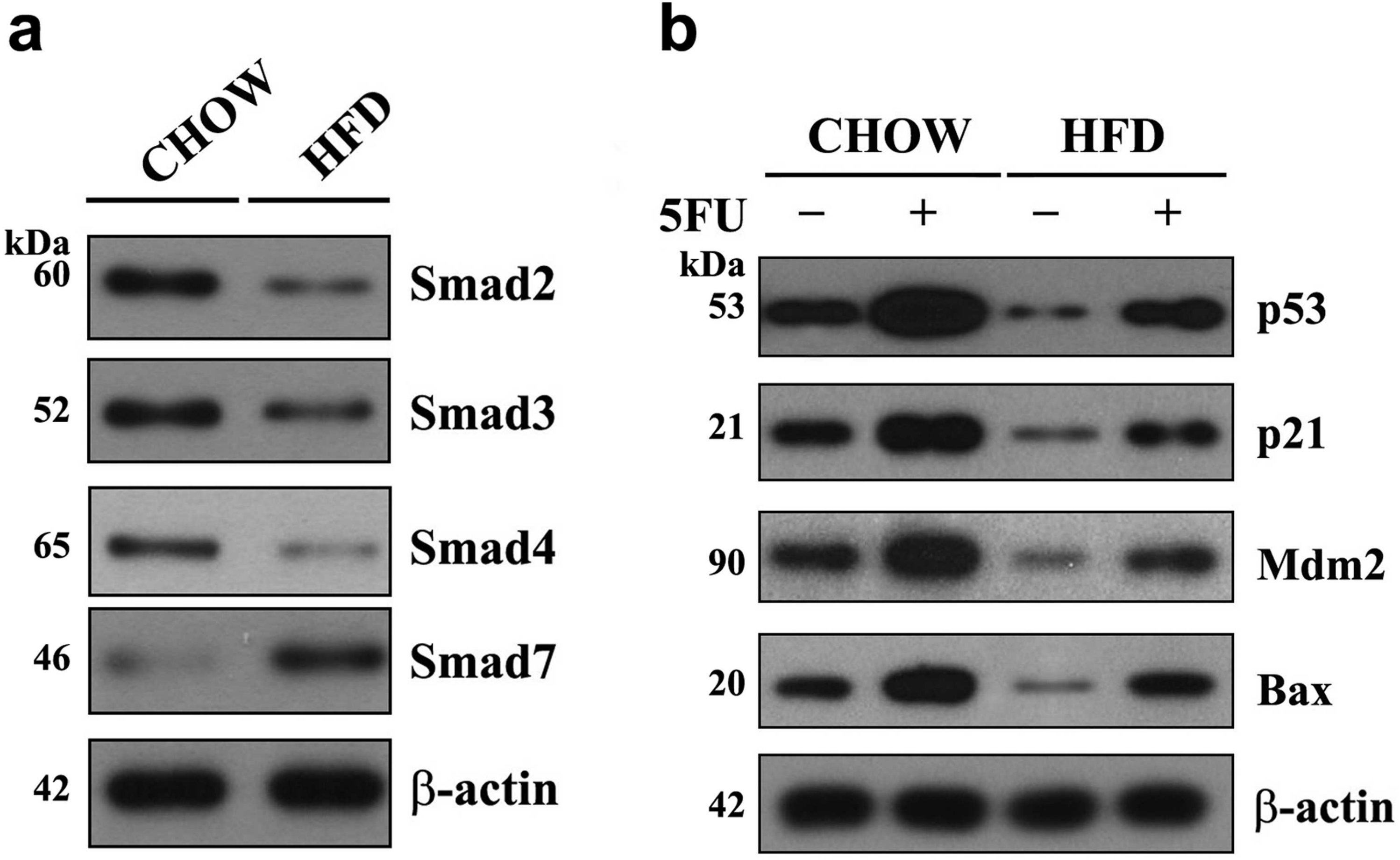

Supplementary Fig.7

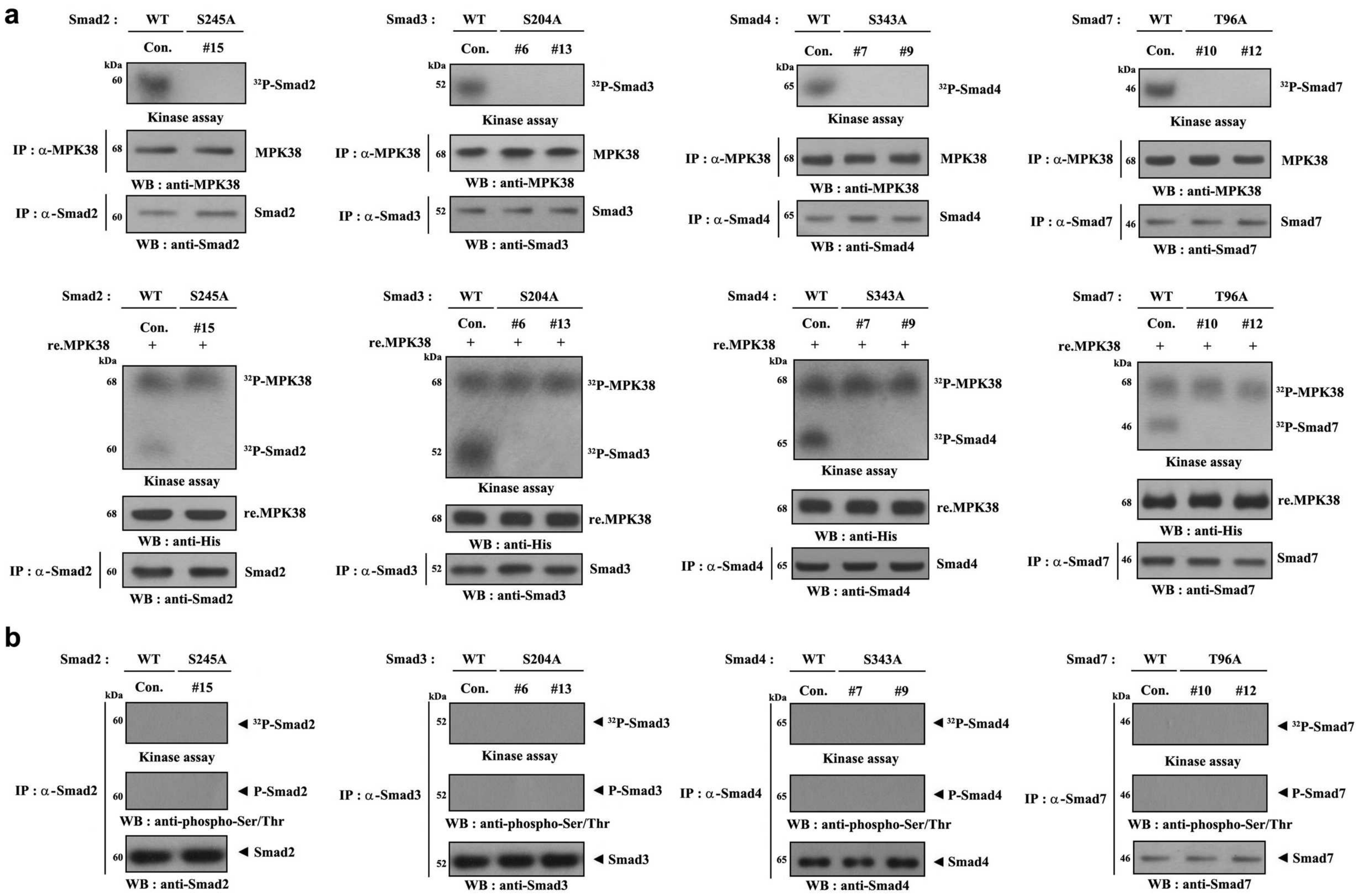

Supplementary Table 1. sg RNAs for genome targeting in vivo

Target	Sequence
Smad2	5`-TTCCCCTGTTAATCATAGCT-3`
Smad3	5`-GTCCCCAGCACATAATAACT-3`
Smad4	5`-TCAGGTAGGAGAGACATTTA-3`
Smad7	5`-GGGCGCCGAGGCGGATCTGA-3`

Supplementary Table 2. Complementary oligos for sgRNA expression plasmids

Target	Sequence
Smad2	Forward 5`-CACCGTTCCCCTGTTAATCATAGCT-3`<br>Reverse 5`-AAACAGCTATGATTAACAGGGGAAC-3`
Smad3	Forward 5`-CACCGGTCCCCAGCACATAATAACT-3`<br>Reverse 5`-AAACATTTATTATGTGCTGGGGACC-3`
Smad4	Forward 5`-CACCGTCAGGTAGGAGAGACATTTA-3`<br>Reverse 5`-AAACTAAATGTCTCTCCTACCTGAC-3`
Smad7	Forward 5`-CACCGGGGCGCCGAGGCGGATCTGA-3`<br>Reverse 5`-AAACTCAGACCTGCCTCGGCGCCCC-3`

Supplementary Table 3. PCR primers for pGEM-T cloning

Target	Sequence
Smad2 (Exon7)	Forward 5`-GAATTCTTGGTATTTAAATTATCTTTG-3`<br>Reverse 5`-TTGGCTATTCATTAGGATCCCTTTCTC-3`
Smad3 (Exon5)	Forward 5`-CAAGGGTATGGGCTAGGCCTTCTCCTG-3`<br>Reverse 5`-CAGGGCTGCAGCTAGAGGGGGTTGCAG-3`
Smad4 (Exon9)	Forward 5`-CCATTTATTTCCTATAGCTCCTGAGTATTG-3`<br>Reverse 5`-TGACTATACAATCAATACCTTGCTCTCTCA-3`
Smad7 (Exon1)	Forward 5`-CCTCGCCTCCTCGCCCCGCATGTTCAG-3`<br>Reverse 5`-CTAGTTCGCAGAGTCGGCTAAGGTGAT-3`

Supplementary Table 4. PCR primers for recombinant adenoviruses

Target	Sequence
Smad2/S245A	Forward 5`-GTAACTATAACGGTCATGTCGTCCATCTTGCCATTCACG-3`<br>Reverse 5`-ATTACCTCTTTCTCCTTATGACATGCTTGAGCAACGCAC-3`
Smad3/S204A	Forward 5`-GTAACTATAACGGTCATGTCGTCCATCCTGCCTTTCACT-3`<br>Reverse 5`-ATTACCTCTTTCTCCCTAAGACACACTGGAACAGCGGAT-3`
Smad4/S343A	Forward 5`-GTAACTATAACGGTCATGGACAATATGTCTATTACGAAT-3`<br>Reverse 5`-ATTACCTCTTTCTCCTCAGTCTAA AGGTTGTGGGTCTGC-3`
Smad7/T96A	Forward 5`-GTAACTATAACGGTCATGTTCAGGACCAAACGATCTGCG-3`<br>Reverse 5`-ATTACCTCTTTCTCCCTACCGGCTGTTGAAGATGACCTC-3`
